# The Short-Term Impact of Animation on the Executive Function of Children Aged 4 to 7

**DOI:** 10.3390/ijerph18168616

**Published:** 2021-08-15

**Authors:** Liheng Fan, Meichen Zhan, Wenjing Qing, Tan Gao, Mengying Wang

**Affiliations:** 1 Institute of Psychology and Behavior, Henan University, Kaifeng 475004, China; 2 Faculty of Education, Henan University, Kaifeng 475004, China; 13253490759@163.com (W.Q.); gaotan666666@163.com (T.G.); mengyingw2021@163.com (M.W.); 3 School of Mathematics and Statistics, Pingdingshan University, Pingdingshan 467000, China; 2378@pdsu.edu.cn

**Keywords:** animation, fantasy, 4–7-year-old children, executive function

## Abstract

Research has shown that animation plays an important role in the development of children’s executive function (EF), and the development of EF components, inhibitory control, working memory, and cognitive flexibility, is asynchronous. Thus, this study explores the developmental trajectories and animation features (fantasy and pacing) that influence each EF component, by examining 218 children aged 4–7. Pretest information, mainly the childhood EF inventory, was provided by parents: child’s age, age of first exposure to animation, animation viewing time on weekdays and weekends, family income, and parents’ education. The children in each age group were randomly divided into four groups to watch animations comprised of different animation features. After watching, their EF were measured by a day-night task, backward digit-span task, and flexible item-selection task. The results showed that the children’s inhibitory control, working memory and cognitive flexibility levels all improved with age. Highly fantastical animations weakened children’s performance on each subsequent EF task. Pacing had no effect on any of the components of children’s EF. An interactive effect on inhibitory control was only found with fantasy in younger children; specifically, high-fantastical animations had a more pronounced short-lived weakening effect on inhibitory control in younger children (4–6 years) compared with low-fantastical animations. Future research should explore the long-term impact of content rather than the form of animation on younger children’s EF.

## 1. Introduction

### 1.1. Executive Function

Executive function (EF) refers to the ability of individuals to consciously control their thoughts and actions [1,2]. The development of EF during childhood is of great significance for the individual’s future life, education, and employment [3,4]. EF can concurrently affect children’s learning and behavior problems [5,6] and can also predict individuals’ behavioral outcomes such as health, wealth, and criminal behavior by the age of 32 [7]. Most of the current studies agree that the three main components of EF are: (a) inhibitory control, namely, the ability to stop focusing on information and/or inhibit dominant responses; (b) working memory (WM), namely, the ability to revise items in working memory by replacing old items with new ones; and (c) flexibility, namely, the ability to transform from one set of rules to another [4,8]. Researchers found that the development of each component of EF is asynchronous [3,9,10]. Moreover, Miyake et al. proposed that EF structure in early childhood might differ from that in later childhood [8]. Therefore, it would be of value to explore the developmental trajectories and factors that influence each component of EF.

### 1.2. The Impact of Television on EF

During early childhood, when the brain has considerable neural plasticity and is rapidly developing, the influence of the environment is particularly salient [11]. While some environmental experiences promote the development of EF, others may hinder it [12,13]. With the development and widespread use of electronic media, children are using such sources more and more often, and often from a very early age [14]. A study of 8950 U.S. preschoolers revealed that they were exposed to TV for approximately 4 h per day [15]. Similarly, a recent Chinese study of 1046 children showed that 70.3% of the sample watched TV daily; 58.7% of those were below the age of two, and children aged 2 to 4 spent at least 2 h daily in front of the TV [16]. In aggregate, these findings indicate that TV may play an important role in the development of children’s EF. The role of TV has consequently become a significant research topic in this field [17].

Research into the effect of TV on children’s EF revealed three types of results, promotion [18,19], no effect [20], and hindrance [21,22,23]. This inconsistency can be ascribed to most studies being of longitudinal, correlational design. In such a design, it is not only difficult to control irrelevant variables, but it is also impossible to determine causality; that is, watching TV can lead to changes in EF, or EF status can affect children’s preference for TV [24,25]. In addition, it is difficult to conduct a long-term study on the TV–EF causal relationship because teachers or parents are unlikely to randomly assign their children to watch TV over an extended period of time. Another way to test causal effect is to test the short-term effect of TV on EF [26]; considering that repeated short-term influences might lead to a long-term effect [24]. Some researchers have begun to use experimental methods to explore the short-term effect of TV on EF [14,27,28].

Pacing is a factor that might affect EF. Pacing refers to the number of times a complete change in scenery occurs [14], such as from a living room to a playground. According to the limited capacity model of TV viewing [29], brain resources for processing information are limited; fast-paced TV programs occupy more of individuals’ cognitive resources, which means that such individuals have insufficient resources to complete subsequent cognitive tasks; thereby, exhibiting worse EF. Some studies found that fast-paced animation can weaken children’s EF. For example, Geist and Gibson [28] asked 4- and 5-year-old children to watch TV programs with different types of pacing. The results showed that children who watched fast-paced programs had significantly lower persistence in subsequent cognitive tasks than children who watched slow-paced programs. However, some researchers found opposite results. For example, Cooper et al. [27] used a computer attention network task (ANT) to measure the effect of watching a 3–5 min short film on the inhibitory control of 4–7-year-old children. The results showed that children who watched a fast-paced short film performed better in terms of inhibitory control.

Lillard et al. [24] proposed that the inconsistent results are probably due to a lack of control over another TV feature, namely, the level of fantasy. Fantasy refers to events that appear frequently in animation but are impossible in reality. It is theorized that human beings have naïve physics, an innate law of governing physical events [30]. Even if it is not innate, such reactions already exist in early life, so even infants have a strong expectation of how events should occur [31]. Accordingly, Lillard et al. [24] suggested that frequently violating these expectations can overload processing because the fantasy information cannot be assimilated into stored patterns. An event that violates the inherent or well-organized representation should be difficult to process and needs more cognitive resources than the actual events. Thus, the presentation of fantasy events might weaken children’s EF. This hypothesis was put to the test in an experiment, and it was found that fantasy weakened children’s performance on EF tasks, while pacing had no effect on EF [24].

### 1.3. The Current Study

The first aim of this study was to investigate the age-related differences in the EF of children aged 4–7 years. Although there is an extensive body of research on EF in early childhood, most studies have focused on a specific age or only focused on the development of children aged 3–6 years, while ignoring the continuity and differences in 7-year-old children’s EF development to a large extent [3]. Some studies suggested that 7-year-olds may be in a stage of rapid development of EF [3,32]. Therefore, the current study assessed the development trail of the three components of 4–7-year-olds’ EF.

The second aim was to explore the short-term impacts of fantasy and pacing on three components of EF. As mentioned above, there are a dearth of relevant studies on the short-term effects of animation on EF, most of which focused on pacing. Lillard and others’ research is the only study to consider both pacing and fantasy [24]. The study, however, had three limitations. First, the EF was considered as a whole, ignoring the uniqueness of each component of EF, and it did not distinguish the distinct influence of different animation features on the specific components of EF. Second, it focused on 4-year-old children exclusively, ignoring the moderating role of age on the animation–EF relation (see more thereon in the next paragraph). Finally, the results required more research for verification. Mounting evidence from neuropsychology shows that the components of EF have different development trails and various connections with differential areas in the prefrontal cortex [33,34,35,36]. Based on the above, the question arises as to whether the pacing and fantasy of animations, as two factors that occupy individual cognitive resources, exert different effects on different components of EF.

The third aim was to determine whether the short-term effects of animation on the three components of EF differed by age. Researchers highlighted the importance of exploring whether some children are more impacted by animations than others [37]. The differential susceptibility to the media effects model (DSMM) posited that not all individuals are affected by media to the same extent and that the influence of media on children’s development is moderated by children’s age; however, there is a lack of research on this topic [38,39]. It is important to determine whether younger children are more vulnerable to the influence of animation and perform worse than older children with respect to EF. The answer would be of great value to discover the sensitive period of animation affecting children’s cognitive development and to implement targeted interventions.

## 2. Materials and Methods

### 2.1. Participants

The participants in this study (*n* = 240) were recruited using a convenience sampling method from two kindergartens and one primary school in Kaifeng, China. The study protocol followed the American Psychological Association’s (APA’s) ethical guidelines and was approved by the principals of the kindergartens and primary school and by the institutional review board of Henan University. Twenty-two children were excluded because their parents did not complete the questionnaires. The final valid sample consisted of 218 children who were approximately 4 (*n* = 54; M = 47.50 months, SD = 5.31 months; 50% girls), 5 (*n* = 55; M = 57.36 months, SD = 4.07 months, 56% girls), 6 (*n* =57; M = 69.90 months, SD = 2.78 months, 49% girls), and 7 (*n* = 52; M = 80.46 months, SD = 4.09 months, 46% girls) years of age.

### 2.2. Procedures

The experiment was conducted with each child individually in an empty room in the child’s school. Children were randomly assigned to one of four conditions at each age level. They viewed the assigned animation on portable computers for approximately 11 min, then completed three EF tests in a random order.

### 2.3. Measures

#### 2.3.1. Animation Materials

Animations varied in terms of their level of pacing and fantasy. Fantastical events refer to impossible transformations in which characters or objects change identity or appearance in an impossible way. The objects or characters are common in everyday life, but the transformations violate the physical laws that govern these entities. The number of fantastical events in the four clips was coded as per Lillard et al. [24]. The occurrence of fantastical events was encoded; if the same event occurred more than once, it was counted only once. The coding was performed by three psychology postgraduate students. First, two coders independently coded every clip, then discussed and resolved any inconsistencies. The third coder then coded 20% of the clips (beginning with a randomly created starting time during the first 80% of the item). The fantasy indexes (fantastical events’ number, events per minute, and the coder’s reliability) are shown in Table 1.

According to the terminology of Lillard and Peterson [14], pacing refers to the number of changes in a complete scene in the animation (for example, from a swimming pool to a playground). The number of scene changes in each animation was counted by two coders. The coders’ consistency reliability was 0.88. The average number of scene changes per minute (rounded to the nearest whole number) was used as an indicator of the pacing of each animation (see Table 1).

#### 2.3.2. Measuring EF

##### Inhibitory Control

The experimental procedure adopted the classic day-night stroop task [40]. Children were shown multiple pictures and asked to report night when they saw a picture of the sun. When they saw a picture of the moon, they were to report day. The experiment was divided into two training sessions and 16 formal sessions (8 cards that depicted daytime scenes (sun pictures)) and 8 cards that depicted evening scenes (moon pictures). To avoid children becoming impatient or visually fatigued, different sun and moon pictures were selected for the test, and each picture was randomly presented using slides. The children scored 0 points for an incorrect response and 1 point for a correct response; scores ranged from 0 to 16 points.

##### Working Memory

The backward digit-span task was used to assess working memory. The experimenter read numbers to the children, who were asked to say them backwards. For example, if the experimenter said “6.5,” the correct response would be 5.6. Children were given practice items with feedback. Once they provided a correct response or after a maximum of four practice items, they moved on to the eight test items, which they continued with until they made three consecutive incorrect responses. One correct response counted as 1 point. If children failed at two digits, 1 point was assigned [32]. The score ranged from 1 to 8 points.

##### Cognitive Flexibility

The flexible item selection task was used to assess cognitive flexibility, with reference to the classic task developed by Jacques and Zelazo [41]. Children were presented with a picture of three shapes with two different dimensions, for instance, color and size, and were first asked to choose the two shapes that matched on one dimension (color) and then to choose the two shapes that matched on another dimension (size). A total of 13 pictures formed the experimental material; 3 that were used for practice and 10 that were used for the formal experiment. The pictures were presented randomly as slides. If the children’s second choice was correct, a score of one point was awarded. The score ranged from 0 to 10 points.

#### 2.3.3. Parent Questionnaires

Two questionnaires were filled out by the parents who had to assess whether there were pre-existing condition differences in their children’s experiences of animations or in EF. One questionnaire inquired parents about the time their children spent in watching animations both on weekends and weekdays, the age at which their children first experienced animations, and the names of animations their children had watched recently. The other questionnaire dealt with the childhood executive functioning inventory (CHEXI) as a pre-test index of EF. Using the same EF task at pre- and post-test was ideal, but EF tasks must involve nonroutine stimuli to engender suitable reactions [42]. When one task was used twice in a short period it loses its novelty; a parent-reporting scale (CHEXI) was therefore used. The scale is consistent with the EF dimension of the present study and is moderately correlated with laboratory EF measurements [43,44]. Parents rated each item on a 5-point Likert rating scale. The total score was calculated by averaging the score for all items, with higher scores indicating greater EF difficulties. Cronbach’s α in the present study was 0.82.

### 2.4. Analytic Strategy

Data were analyzed in two steps; in the first step, a pretest analysis was conducted, using multivariate ANOVA, to analyze differences in the four groups of animation characteristics on pre-test variables for children, and a Bonferroni test was used for post hoc tests. A formal analysis was conducted in the second step, with the three components of EF as dependent variables, and age, pacing, and fantasy as independent variables. The analysis also included a univariate ANOVA using the significant variables from the pretest analysis as control variables and Bonferroni tests to test for differences in the main effects and interaction effects. Statistical significance was defined as a *p*-value of <0.05, and critical significance was defined as a *p*-value of <0.08.

## 3. Results

### 3.1. Preliminary Analyses

The four groups of animation types were first compared on the children’s background variables. Table 2 shows the descriptive statistics of the four groups. The analysis indicated that no significant group differences were found with respect to gender (F(3, 214) = 0.05, *p* = 0.98), family income (F(3, 214) = 1.62, *p* = 0.91), parental education (F(3, 214) = 0.36, *p* = 0.78), age of first exposure to animations (F(3, 214) = 0.52, *p* = 0.67), amount of animation viewing on weekdays (F(3, 214) = 2.58, *p* = 0.08), and the pre-test EF scores (F(3, 214) = 0.66, *p* = 0.58), were not significant (n.s.). These variables were not considered further. However, there was a significant difference in animation viewing on weekends among children in the four groups, (F(3, 214) = 3.09, *p* < 0.05, *η^2^* = 0.04). Post hoc tests indicated that children in the high-fantastical and fast-paced group (MD = 2.1, SE = 0.98) viewed a critically (*p* = 0.07) longer amount of animations on weekends than children in the highly fantastical and slow paced group (MD = 1.63, SE = 0.96). Therefore, the duration of animation viewing on weekends was controlled as a covariate in subsequent analyses.

### 3.2. Main Analyses

Table 3 shows the descriptive statistics of the three components of EF according to age, fantasy, and pacing. A 2 (pacing: fast or slow) × 2 (fantasy: high or low) × 4 (age) analysis of variance (ANOVA) between subjects was used, controlling the factor of animation viewing time on weekends. The main effects of age were significant on three EF components, namely inhibitory control, F(3, 201) = 18.87, *p* < 0.001, *η*^2^ = 0.22; working memory, F(3, 201) = 29.47, *p* < 0.001, *η*^2^ = 0.31; and cognitive flexibility, F(3, 201) = 7.55, *p* < 0.001, *η*^2^ = 0.10.

Post hoc tests revealed that, for inhibitory control (M_4_ = 12.13, SD_4_ = 2.81; M_5_ =13.05, SD_5_ = 2.23; M_6_ = 13.30, SD_6_ = 1.57; M_7_ = 14.62, SD_7_ = 1.19) and cognitive flexibility (M_4_ = 7.52, SD_4_ = 1.92; M_5_ = 8.25, SD_5_ = 2.14; M_6_ = 8.18, SD_6_ = 1.70; M_7_ = 9.06, SD_7_ = 1.27), the scores of the 4-year-old group were significantly lower than those of the 5-, 6-, and 7-year-old groups; the 7-year-old group scored significantly higher than the other three age groups, and there were no significant differences between the 5- and 6-year-old groups. Pairwise differences for working memory were significant between all age groups, namely, the 4-year-old group (M_4_ = 2.30, SD_4_ = 0.82) scored significantly lower than the 5- (M_5_ = 2.69, SD_5_ = 0.92), 6- (M_6_ = 3.04, SD_6_ = 0.80), and 7-year-old groups (M_7_ = 3.71, SD_7_ = 0.94); the 5-year-old group scored significantly lower than the 6- and 7-year-old groups; and the 6-year-old group scored significantly lower than the 7-year-old group.

The main effects of fantasy were significant for all three EF components: F_IC_(1, 201) = 85.88, *p* < 0.001, *η^2^* = 0.30; F_WM_(1, 201) = 76.64, *p* < 0.001, *η^2^* = 0.28; F_CF_(1, 201) = 53.48, *p* < 0.001, *η^2^* = 0.21. Post hoc tests revealed that compared with those who watched low-fantastical animations, children in the high-fantastical group performed worse on inhibitory control (M_low_ = 14.33, SD_low_ = 1.34; M_high_ = 12.18, SD_high_ = 2.40; *p* < 0.001), working memory (M_low_ = 3.36, SD_low_ = 0.94; M_hihg_ = 2.48, SD_high_ = 0.87; *p* < 0.001), and cognitive flexibility (M_low_ = 9.04, SD_low_ = 1.23; M_high_ = 7.44, SD_high_ = 2.04; *p* < 0.001).

**Figure 1:**
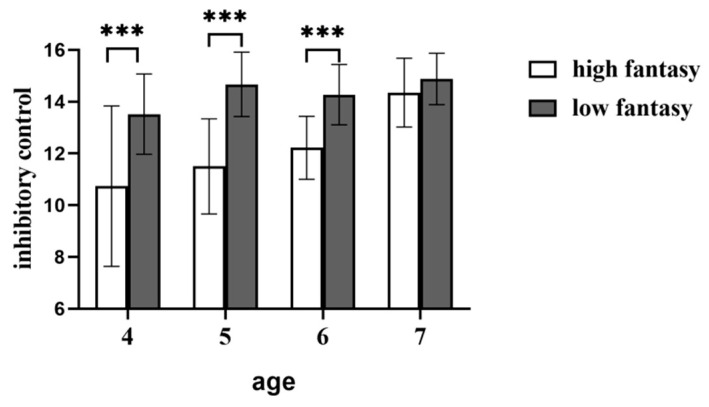
Inhibitory control performance for the different levels of fantasy and age groups (*** *p* < 0.001).

Only one interaction effect was significant, namely age × fantasy on inhibitory control, F(1, 201) = 6.10, *p =* 0.001, *η^2^* = 0.08 (see Figure 1). A simple effects analysis showed that among the 4- (M_high_ = 10.74, SD_high_ = 3.10; M_low_ = 13.52, SD_low_ = 1.55), 5- (M_high_ = 11.50, SD_high_ = 1.84; M_low_ = 14.67, SD_low_ = 1.24), and 6-year-old groups (M_high_ = 12.22, SD_high_ = 1.22; M_low_ = 14.27, SD_low_ = 1.17), high-fantastical animations were associated with worse inhibitory control than low-fantastical animations. There was no significant difference in inhibitory control between 7-year-old children who watched animations with different fantasy levels (M_high_ = 14.35, SD_high_ = 1.33; M_low_ = 14.88, SD_low_ = 0.99). It is therefore evident that, compared with 7-year-old children, the inhibitory control of children aged 4 to 6 years is more likely to be affected by the level of animation fantasy.

## 4. Discussion

This study explored the effects of age, fantasy and pacing of animations on 4–7-year-old children’s performance in the three EF components. After adjusting for various confounders, the empirical results revealed that there were different age development trajectories among the three EF components, and watching an 11 min episode of a high-fantastical animation immediately impaired children’s EF relative to watching a low-fantastical animation. However, no effect of animation pacing on EF was found. Further, the only significant interaction effect was between age and fantasy on inhibitory control.

The results show that each component of EF improved well with age development, but there were age differences in the development of different components of EF. Specifically for inhibitory control, there were two clear developmental periods, namely 4–5-years old and 6–7-years old. Some researchers have suggested that the age of 4-years old is the key period for the development of inhibitory control [45,46]. Three-year-old children were not selected for this study, and the role of the age of 4 years in individual development could therefore not be determined. However, Xie found that inhibitory control develops rapidly between 4.5 and 5 years [47]. In contrast, Brocki and Bohlin found that the age of 6–7 years represented a period of rapid growth in inhibitory control [48], and Best and Miller suggested that the age of 7 years was the key period for individual inhibitory control development [3]. Based on these results, 4–5 years and 6–7 years of age appear to be significant developmental stages for individual inhibitory control. There were significant differences on working memory among all age groups, which further support the linear development trend in working memory from preschool to adolescence [3]. Regarding cognitive flexibility, we found that 4–5 and 6–7 years of age represent periods of rapid development, which is consistent with Best and Miller’s research [3] on 4–5-year-olds and Sun’s research [49] on 6–7-year-olds. Combined with the age differences in the three components of EF, we suggest that 5 and 7 years of age are critical stages of children’s EF development; however, further research is needed to verify this suggestion.

The results indicate that fantastical content in animation significantly impacted the three components of the Chinese children’s EF. There are three possible reasons for this finding. The first relates to limitations of cognitive resources. Carey suggested that even very young children had a strong expectation about how things should happen [31]. Events that do not conform to children’s expectations may overload information processing and weaken the children’s subsequent cognitive behavior [50]. Second, the attention and processing of fantastical events might deplete the neurotransmitters in the lateral prefrontal cortex and reduce children’s availability for subsequent cognitive tasks [51]. Therefore, short-term exposure to highly fantastical animation would lead to worse performance on subsequent EF tasks. Third, after children watched engaging animation programs, they need time to shift their attention system from bottom-up processing to top-down processing [24]. According to the TV processing model, TV stimuli are first focused, then encoded, processed, and finally stored [52]. Performing EF tasks depends on these same processes, but in different ways. Viewing animation involves a bottom-up process of orientation and reminding. Watching fantastical events attracts more attention and triggers more directional reactions. However, over short periods of time, the attentional system might have insufficient time to shift from bottom-up to the top-down processing required for EF, thereby leading to temporarily diminished EF.

No effect of pacing on children’s EF was found, which may reflect a general trend toward increased pacing of animations [53]. According to Xing et al., fast pacing has become the main form of children’s animations [19]. Thus, the short-term effect of pacing may be relatively weak. In addition, Bushman and Miller found that the stimuli presented with fast pacing only involve sensory processing, not prefrontal cortical processing [54]. Thus, watching fast-paced animation does not involve prefrontal neurotransmitters, and hence did not affect the subsequent EF tasks in the current study. This result is consistent with short-term impact research [24] and recent long-term correlational research [19,55]. Thus, it appears that the pacing of animations does not affect the development of children’s EF, neither in the short term nor in the long term. Future research can focus more on the content characteristics of animations, such as fantasy, humor, authenticity, and so on.

There was a significant age × fantasy interaction on inhibitory control whereby, compared with watching low-fantastical animations, watching high-fantastical animations led 4–6-year-old children to exhibit worse inhibitory control. This may be because both fantastical events and inhibitory control tasks require the participation of the anterior cingulate cortex (ACC). The fantastical events did not conform to the physical properties or rules that govern entities in the real world. Hence, processing these events needed to activate the ACC, which is related to the prefrontal cortex (PFC) [56]. The inhibitory control task we used in this study was a Stroop task, which also needed the participation of the ACC [57]. Accordingly, watching highly fantastical animations consumed more cognitive resources in the ACC. This led to diminished performance in the subsequent inhibitory control task due to a temporary lack of cognitive resources. Furthermore, the strength model of self-control posits that all self-control requires the same resources, and that the consumption of resources in one domain necessarily affects available resources in another domain [58]. In contrast to viewing low-fantasy animation, when viewing high-fantasy animation, children need to constantly inhibit their existing real-world rules and knowledge to conform to the illusory world created by the animation, thereby hindering their subsequent normal performance on the inhibitory control task.

From one perspective, 7-year-old children did not exhibit this characteristic because the inhibitory control of 7-year-old children is equivalent to that of 12-year-old children [59]. Hence, the demands on cognitive resources were modest when they completed the inhibitory control task, and the highly fantastical events had a weaker impact. From another perspective, the performance characteristics might be related to the development of the PFC. A right PFC advantage of 4–6-year-old children when completing an inhibitory task begins to transform to a left PFC advantage at the age of 7 [60]. As such, participation of language and other aspects of cognition represented by the left PFC reduced the resources required for the inhibitory control task, leading to a failure of 7-year-olds to exhibit this negative effect. Young children (4–6 years old) were more likely to be restricted by the development of their cerebral cortices and cognitive functions, and to consume more cognitive resources when watching highly fantastical animation, as a result of which they then exhibited poorer inhibitory control.

The age interaction was not indicative of cognitive flexibility and working memory. This might be related to the developmental characteristics of two EF components. First, cognitive flexibility and working memory are among the more complex and more late-developing components of EF compared with early-developing inhibitory control [61]. Second, cognitive flexibility requires the joint participation of inhibitory control and working memory [3], while the working memory task in this study was a reverse digit span task, which is a complex task that requires the participation of multiple cognitive functions, such as inhibitory control [3]. Although children’s cognitive flexibility and working memory develop rapidly between the ages of 4 and 7, the participants were still in the primary stages of brain development and the initial stages of cognitive development [62]. In this way, viewing animations, whether of high or low fantasy, prevented higher cognitive activities that require more cognitive resources from proceeding smoothly because of the cognitive resources they took up. Hence, age differences in animation effects on these two EF components became insignificant. Considering that in real life, children under 7 years are the main targets of animations, and such animations are characterized by fantastical components, the short-term weakening effect of highly fantastical animations on children’s inhibitory control requires further research.

### Limitations

There are several limitations of the study that should be mentioned. In the first instance, this study assessed only the short-term effects (i.e., over an 11 min period) of animations on EF. It is unclear whether watching animations impacts EF in the long term, which needs further longitudinal studies to explore. Second, this study only used three kinds of tasks to measure the three components of EF, which could not comprehensively represent all aspects of each component. Future research should consider examining different EF tasks that allow for a comprehensive measurement of EF. Finally, although this study divided age groups by one year, consistent with previous studies, this might not reflect the characteristics of the rapid development of children’s EF. In the future, more fine-grained age divisions (e.g., 6 months) can be assessed to explore the developmental trajectory of EF more clearly and sensitively.

## 5. Conclusions

The three components of EF reflected different developmental trajectories from 4 to 7 years of age. Watching highly fantastical animations weakened the children’s subsequent EF. In addition, compared with low-fantastical animations, watching high-fantastical animations had a greater short-term negative impact on the inhibitory control of younger children. The results suggest that future research should distinguish the different components of EF and further explore the mechanisms by which highly fantastical animations influence the inhibitory control of younger children.

## Figures and Tables

**Table 1:** Pacing and Fantasy Characteristics of Cartoon Programs.

Program	Length	Pacing (Scenes per Minute)	Number of Fantasy Events (Reliability %)	Fantasy Rate (Events per Minute)
SpongeBob	9:59	35	3.00 (90%)	30
Tom and Jerry	11:10	8	3.22 (83%)	36
Boonie Bear	10:00	38	0.33 (100%)	3
Big Head Son and Little Head Father	11:13	7	0.36 (100%)	4

**Table 2:** Descriptive Statistics Among the Four Groups (M ± SD).

	High Fantasy		Low Fantasy		Range
	Fast Paced (*n* = 51)	Slow Paced (*n* = 57)	Fast Paced (*n* = 56)	Slow Paced (*n* = 54)	
Gender	0.49 ± 0.51	0.49 ± 0.50	0.52 ± 0.50	0.48 ±0.50	[0.43, 0.56]
Family income	3.73 ± 1.02	3.35 ± 1.48	3.36 ± 1.45	3.46 ± 1.30	[3.30, 3.65]
Parental education	3.61 ± 0.90	3.56 ± 0.82	3.66 ± 1.12	3.48 ±0.89	[3.45, 3.70]
Age of first exposure to animation (months)	24.76 ± 13.00	26.19 ± 11.00	23.95 ± 12.60	26.52 ± 13.44	[23.63, 27.06]
Animations viewed on workdays (hours per day)	1.98 ± 1.16	1.68 ± 1.11	2.21 ± 1.07	1.76 ± 1.12	[1.78, 2.07]
Animations viewed over weekends (hours per day)	2.10 ± 0.98	1.63 ± 0.96	2.02 ± 0.98	1.70 ± 0.92	[1.72, 2.00]
Pre-EF score	2.65 ± 0.53	2.56 ± 0.64	2.57 ± 0.60	2.70 ± 0.61	[2.54, 2.69]

Gender:0—female, 1—male. Family income means the annual income of a family: 3—15,000 to 30,000 RMB; 4—30,000 to 60,000 RMB. Parental education: 3—associates degree; 4—bachelor’s degree.

**Table 3:** Scores of the three components of EF according to age, fantasy, and pacing (M ± SD).

	Inhibitory Control				Working Memory				Cognitive Flexibility			
Age	High Fantasy		Low Fantasy		High Fantasy		Low Fantasy		High Fantasy		Low Fantasy	
	Fast paced	Slow paced	Fast paced	Slow paced	Fast paced	Slow paced	Fast paced	Slow paced	Fast paced	Slow paced	Fast paced	Slow paced
4	10.36 ± 3.30	11.29 ± 2.89	13.79 ± 1.58	13.29 ± 1.49	2.07 ± 1.00	1.93 ± 0.62	2.86 ± 0.60	2.43 ± 0.65	6.79 ± 2.23	6.64 ± 1.95	8.57 ± 1.34	8.00 ± 1.30
5	11.29 ± 2.02	11.93 ± 1.59	14.08 ± 1.32	15.21 ± 0.89	2.29 ± 0.83	2.07 ± 0.62	3.23 ± 0.83	3.21 ± 0.80	7.57 ± 2.31	7.21 ± 2.86	8.92 ± 1.26	9.36 ± 0.75
6	12.14 ± 1.17	12.29 ± 1.27	14.73 ± 0.96	13.79 ± 1.25	2.43 ± 0.51	2.57 ± 0.51	3.53 ± 0.83	3.50 ± 0.65	7.36 ± 1.55	7.21 ± 1.59	9.47 ± 0.92	8.57 ± 1.70
7	13.67 ± 1.68	14.33 ± 1.28	14.56 ± 1.42	14.83 ± 1.20	3.17 ± 0.79	3.44 ± 0.86	4.11 ± 1.08	4.00 ± 0.77	7.61 ± 1.61	8.33 ± 1.61	9.50 ± 0.79	9.50 ± 0.86

## Data Availability

The data presented in this study are available on request from the corresponding author.
